# Wyethia Helenioides, with Additional Provings

**Published:** 1898-09

**Authors:** J. M. Selfridge

**Affiliations:** Oakland, Cal.


THE
HOMŒOPATHIC PHYSICIAN,
A MONTHLY JOURNAL OF
HOMŒOPATHIC MATERIA MEDICA AND CLINICAL MEDICINE.

"If our school ever gives up the strict inductive method of Hahnemann, we are lost, and deserve only to be mentioned as a caricature in the history of medicine."—CONSTANTINE HERING.

Vol. XVIII. SEPTEMBER, 1898. No. 9.

### WYETHIA HELENIOIDES, WITH ADDITIONAL PROVINGS.

J. M. SELFRIDGE, M. D., OAKLAND, CAL.

There is probably no State in the Union where there is a greater number of valuable remedies to be found than in the State of California. These remedies are waiting to be proven by those of us who have sufficient enthusiasm in medicine, and who are willing to take the trouble and make what sacrifice is necessary to accomplish so desirable a result. I know it has been said that we have too many remedies which have not been properly proven. While this is doubtless true, it is equally true that many of the new remedies which have been introduced within the memory of some of us, are absolutely indispensable in the treatment of certain forms of disease.

There is another reason why these California remedies should become a part of our armamentarium. It is claimed by Teste and others that where certain forms of disease prevail there, or in that vicinity, the curative remedy may be found.

Again, it has been said that there is a remedy somewhere in nature for every ill to which flesh is heir. Whether this be true or not, we know there are diseases which, so far as we are aware, are incurable, for the simple reason that we know of no remedy that will control the abnormal conditions. This being true, the incentive ought to be sufficiently great to urge us forward in the line of knowing more than we now know of the wealth of those remedies that lie at our very doors. All we know of these drugs, so far, are mere hints which have been given us by the older inhabitants of the coast. Thus, the Eriodyction Californicum, or "Yerba Santa," has been suggested for the cure of "poison oak," and for certain bronchial affections. A partial proving of it was made some years ago, under the supervision of the late Dr. Pease, which can be found in Allen's *Encyclopedia,* Vol. IV, page 218.

The Micromeria Douglassii, or "Yerba Bueno," is another plant which should be proven. Many years ago a friend of mine was suffering with a series of boils, when an old "Spanish woman" directed him to make a tea of this plant. This he did, and cured his boils; but, as the tea had an agreeable taste, he continued to drink it, believing, as some do, that if "little was good, more was better," until finally he became so weak he could not continue his work.

It was one of these hints that induced me some years ago to make a proving of Wyethia Helenioides, or "poison weed." Like many other provings, it was only partial. A schema of it was published in Allen's *Encyclopedia*, Vol. X, page 168.

The proving then made was the following:

The symptoms here recorded were observed by seven men and two women.

The green root in substance or the mother tincture was used by each prover. When the tincture was used the dose varied from five to forty drops.

The plant has been used by unprofessional people in some of the interior towns of this State for coughs and colds, under the name of "poison weed."

The taste of the root is herbaceous, aromatic, and slightly bitter, leaving a sweetish sensation in the mouth.

The symptoms here recorded are given in the order of their occurrence as nearly as could be ascertained. No symptoms were manifested for some minutes after chewing the root or swallowing the tincture.

SYMPTOMS.

Sense of weight in the stomach, as if something indigestible had been eaten; belching of wind alternating with hiccoughs; mouth feels as if it had been scalded; sensation of heat down the œsophagus into the stomach, worse while eating; dryness of the fauces; constant desire to clear the throat by hemming; increased flow of tough, ropy saliva; throat feels swollen; epiglottis dry and has a burning sensation; constant desire to swallow saliva to relieve the dryness, yet affording no comfort; swallows with difficulty; pricking, dry sensation in posterior nares, sensation as if something were in the nasal passages; an effort to clear them through the throat affords no relief; the uvula feels elongated; dry, hacking cough, caused by tickling of the epiglottis; pain in the back, which extends to the extreme point of the spine; pain in the left ovary, shooting down to the knee; pain in the right arm, with stiffness of the wrist and hand; severe headache; rush of blood to the head; dizziness; pain in the forehead over the right eye, at first sharp, followed by a sensation of fullness; itching in the right ear; cold sweat over the whole body, which soon dries off, and again comes and goes as if by flashes; nausea and vomiting; burning sensation in the bronchial tubes; sharp pain just below the ribs on the right side, deep seated, followed by soreness; passages of the bowels previously light colored, irregular and constipated, become dark-colored, regular, and soft; passage loose, diarrhœic, dark-brown color, came on in the night and lasted five days; itching of the anus; great constipation accompanied with hæmorrhoids, not bleeding, never had them before or since (three provers); passages small, dark brown, look as if burned; feels weak and nervous, uneasy, apprehensive, as if some dire calamity were about to occur; pain and bearing down in right side; leucorrhœa; menses appeared for the first time in over a year, since the birth of last child; color purple and scanty, with great pain; feel very weak, as a person feels after a severe illness; unable to make much exertion; the least exercise causes perspiration; slowness of pulse, decreased in ten hours from 72 to 58 beats per minute. All the symptoms worse in the afternoon.

REMARKS:—It will be observed that Wyethia affects the brain and nervous system, the mucous membrane of the throat and bronchi, the liver and portal vessels, and also the reproductive organs of the female. It has proved beneficial in dry asthma and chronic follicular pharyngitis. It has relieved the dryness of the pharynx and the burning of the epiglottis. It has repeatedly removed the inflammation of the mucous follicles of the pharynx, even when sufficiently numerous to give the membrane "a granular or mamillated appearance." It has proved beneficial in cases of irritable throats of singers and public speakers.

Two years ago an attempt was made to secure additional symptoms, which are given below in the language of the provers, who at that time were members of "*The Organon* and Materia Medica Club of the Bay Cities."

At the time of proving the potency and the drug were unknown to the provers.

### FIRST PROVER.*

June 9th, 1896.—Began taking Wyethia, of which I took a drop in a teaspoonful of water before each meal. First dose 7.35 (did this for four days); 7.45, feels in nose as if about to sneeze; 7.50, sitting quietly, a momentary pain on inside of right foot from instep to the sole; 8.35, stretching

* Dr. McNeil took the first decimal dilution. (S.)

and yawning, itching on the left side of the chin; 4.10 P. M., dry sensation in throat, although mucous is abundant; 5.30 P. M., sensation of dryness and tickling on the edges of the eyelids, such as I felt when a stye was about to appear, sensation of dryness in throat; 5.35 P. M., a small itching spot on right side of neck; 8.00 P. M., dryness in throat, with abundant mucus.

June 7.—7.30 A. M., throat sore; 8.35, tingling in right foot when standing; 11.00, while in church, sensations of formications in eyelids, with lachrymation; 11.25, pain in the right testicle; 3 P. M., despondent; 6 P. M., pain on top of right shoulder midway between neck and point of shoulder, motion does not affect it.

June 8.—Before breakfast, lips feel dry, back of throat (posterior wall of pharynx) sore, increased flow of tasteless saliva; 10.30, pain in left ear, itching in left external canthus; 1.30 P. M., mouth full of sweetish saliva; at lunch bit tongue severely; 9.30 P. M., mouth feels dry and as if scalded, with desire to drink frequently in order to moisten it.

June 9.—Scalded mouth continues.

June 12.—6 A. M., lips feel scalded and swollen.

June 17.—Itching in rectum.

July 4.—10 A. M., headache left anterior part of brain, as if radiated from left inner canthus; 12.30, headache in left occipital protuberance. For several nights waken frequently and too early in the morning, without any disagreeable consequences.

July 7.—A sore hang-nail on third finger of left hand.

(Signed) A. McNEIL.

### SECOND PROVER.*

June 5.—Began at 1 P. M., taking a drop before each meal.

June 6.—Depressed all forenoon, languid feeling of mind and body; despondent almost to desperation; irritable, cross,

* Dr. Underwood took the fifteenth decimal dilution. (S.)

easily angered about trifles; melancholy about the future, with no reason for it; seemed that I was forsaken by all my friends and was on the verge of insanity; bodily uneasiness, unfitting me for any work; felt that I could "*fall all down in a heap;*" muscles seemed to refuse to respond to the will.

June 7.—Entire incapacity for mental work; could not follow a line of thought twenty seconds; forehead cold to touch, with heavy feeling over the eyes as though the skin and flesh of forehead would come down over the eyes; intense drowsiness all day, worse after meals; irresistible sleepiness after lunch; accustomed cup of coffee was not relished.

June 8.—Dreams were vivid and real; was discovered talking in my sleep; the thoughts and work of previous day were on my mind on waking, as though I had not gone to sleep.

June 9-10.—Aversion to company; did not wish to see any one, not even intimate friends; great aversion to my work; had to punish myself to even visit a patient; quarrelsome, impatient, irritable.

(Signed) M. F. UNDERWOOD.

THIRD PROVER.*

June 8, 1896.—Commenced taking remedy given by Dr. J. M. Selfridge, one drop three times a day before meals.

June 13.—After a restless night awakened at 7.30 A. M., with severe, sharp pain in right tonsil; throat felt swollen and sore; tonsil red and inflamed; glands on right side of neck swollen and sore to touch.

At 9.30 neuralgic pains commenced in left arm and hand, then in back, limbs, and all over the body; skin felt sore to touch. Was quite ill all day, with no appetite whatever.

At 7.30 P. M. commenced to feel chilly; upon the slightest movement chills would creep up the back, with increase of pain; grew colder and colder, was very ill, and went to bed. At 9.30 fever commenced, with desire for food; head very

* Dr. Martin took the thirtieth decimal dilution. (S.)

hot; cheeks very red and burning; temperature 102 degrees, but still felt very chilly. Passed a very restless night, with chill, fever, and sweat all at the same time, with constant twinges of pain all over the body, particularly in back and limbs; could not bear the slightest touch.

June 14.—Temperature 101½ degrees at 8 A. M. Right tonsil and glands of neck still very sore, in fact, worse; pains over body less, though back quite sore and lame; felt very weak and unable to remain out of bed.

Still continued the remedy. All symptoms gradually improved, and was entirely well in a few days.

June 20.—Stopped taking the remedy upon advice of Dr. Selfridge.

June 21.—Very depressed, both mentally and physically; menses comenced at 2.30 P. M., with slight uterine pain. Retired at 10 o'clock, when the pain became intense and burning. Suffered all night, the pain being constant, though increasing in paroxysms with sensation as if the uterus expanded in order to keep all the pain within its walls. Could distinctly outline the contour of the uterus. Never had just such a pain before.

June 22.—Pain much better, but still a paroxysm every little while. Felt very weak all day, and mentally depressed.

When menses ceased, observed no further symptoms.

July 4.—Commenced remedy again.

July 18.—At 11 A. M., comenced to feel chilly, with aching pains all over the body, which gradually grew worse until 12 o'clock, when a most severe chill took place; shook all over; aching over body and headache intense. Took no more of the remedy, went to bed, and as I was growing worse, was given Aconite at one o'clock. There was great thirst for ice water during the entire chill, which lasted until 2.30 P. M., when fever came on; temperature 101 degrees, no thirst. In about fifteen minutes commenced to sweat. Temperature at 4 o'clock, 100 degrees; still sweating. At 10 P. M. menses commenced; no uterine pain, but still aching all over body, which continued all night, preventing sleep; pains worse in limbs and back; at times jerking in character, making me start with every twinge; profuse sweating all night.

July 19.—Very weak; aching still continued, but less; cords of neck, right side, quite painful; passed a restless night, still sweating profusely.

July 20.—Much better, but still very weak; some aching and sweating; did not go to sleep until 3 A. M.; was nervous and restless.

July 21.—Much improved in every way, and was all right in a day or two. Did not take any more of the remedy.

July 26.—At 1.30 P. M. commenced to feel chilly, with intense headache and aching all over the body. The chilliness rapidly increased until at 2 o'clock had a worse chill than ever, which lasted until 4 o'clock, when fever came on, temperature soon reaching 103 degrees; sweating commenced almost simultaneously with the fever; headache was the most prominent symptom, which was terrific; intense, congestive headache; eyes extremely sensitive; bones of the face sensitive to touch; could not move the head a hair's breadth without intense agony; thought I should go mad from the intensity of the pain. This lasted until 10.30, when there was a sensation of faintness, due evidently to lack of food, which passed away after eating some cream toast; the headache then also began to grow less, and I passed a fairly good night.

July 27.—Was much better, but was too nervous to remain in bed; felt very weak all day; retired early, but did not sleep a moment all night long.

July 28.—Arose at 6 A. M.; was weak and dizzy all day; had to lie down every little while. Slept well this night.

Have been feeling fairly well ever since.

(Signed) ELEANOR F. MARTIN.

AUGUST 7th, 1896.

